# Contamination Characteristics of 21 PFAS in Shellfish and Crustaceans of Zhejiang Province and Exposure Risk Assessment for Adult Dietary Consumers

**DOI:** 10.3390/md23090359

**Published:** 2025-09-15

**Authors:** Hexiang Zhang, Haoyi Zhang, Ronghua Zhang, Dong Zhao, Bing Zhu, Xiaojuan Qi, Lili Chen, Jiang Chen, Jikai Wang, Yibin Zheng, Zhewei Feng

**Affiliations:** 1 Zhejiang Provincial Center for Disease Control and Prevention, Hangzhou 310051, China; hxzhang@cdc.zj.cn (H.Z.); zhyxmu3475@stu.xmu.edu.cn (H.Z.); rhzhang@cdc.zj.cn (R.Z.); dzhao@cdc.zj.cn (D.Z.); bzh@cdc.zj.cn (B.Z.); xjqi@cdc.zj.cn (X.Q.); llchen@cdc.zj.cn (L.C.); jchen@cdc.zj.cn (J.C.); jkwang@cdc.zj.cn (J.W.); 2 School of Public Health, Xiamen University, Xiamen 361005, China; 3 Hangzhou Gongshu District Center for Disease Control and Prevention (Hangzhou Gongshu District Health Supervision Institution), Hangzhou 310022, China

**Keywords:** perfluoroalkyl and polyfluoroalkyl substances (PFAS), bivalve shellfish, crustaceans, exposure assessment

## Abstract

This study investigated the occurrence, sources, and health risks of 21 per- and polyfluoroalkyl substances (PFAS) in commercially available shellfish and crustaceans from Zhejiang Province, China. Among the 306 samples analyzed, 87.9% contained at least one detectable PFAS. Perfluorooctanoic acid (PFOA) was the most frequently detected PFAS (64.7%), followed by perfluorooctanesulfonic acid (PFOS) (53.8%), perfluorononanoic acid (PFNA) (52.9%), and perfluorodecanoic acid (PFDA) (50.0%). The total PFAS in shellfish and crustaceans ranged from ND to 0.86 to 173 ng/g wet weight, with a median of 4.11 ng/g ww; the median concentration of total PFAS followed this order: marine crustaceans > fresh-water crustaceans > bivalves. Estimation of the human intake of adult consumers, the estimated daily intake (EDI) of Σ_21_ PFAS ranged from 0.01 to 15.7 ng/kg bw/day; 0.31% of the adult study population had Σ_4_PFAS exposure levels resulting in Hazard Quotient (HQ) values > 1, which may represent a potential public health concern for these individuals. Long-term exposure risks for specific PFCAs such as perfluoroundecanoic acid (PFUdA) and perfluorotridecanoic acid (PFTrDA) merit concern.

## 1. Introduction

Perfluoroalkyl and polyfluoroalkyl substances (PFAS) are a large and diverse group of man-made chemicals characterized by their unique physicochemical properties and broad range of applications. PFAS refer to compounds containing at least one of the following three structural configurations: (1) R-(CF_2_)-CF(R′)″, where both CF_2_ and CF moieties are saturated carbons; (2) R-CF_2_OCF_2_-R′, where R and R′ may be F, O, or saturated carbon; (3) CF_3_C(CF_3_) R′ R″, where R′ and R″ may be F or saturated carbon. The total number of PFAS may reach 13,054 distinct compounds [1]. Characterized by hydrophilic functional groups and hydrophobic alkyl side chains, PFAS exhibit fire resistance, high stability, and persistence [2]. Consequently, they are extensively utilized in foam fire extinguishers, lubricants, surfactants, textile additives, and chromium mist inhibitors in electroplating processes [3]. Currently, compounds of significant concern include pentadecafluorooctanoic acid (PFOA), perfluorodecanoic acid (PFDA), perfluorotridecanoic acid (PFTrDA), and particularly perfluoroundecanoic acid (PFUdA) from the perfluorocarboxylic acids (PFCAs) category; perfluorooctane sulfonate (PFOS) and henicosafluorodecanesulphonic acid (PFDS) from the perfluorosulfonic acids (PFSAs); and newly emerging PFAS such as 4,8-Dioxa-3H-perfluorononanoic acid (ADONA) [4,5].

PFAS exhibit multi-organ toxicity and carcinogenicity, and their environmental persistence and mobility lead to widespread distribution across various environmental media. PFAS have broad toxic effects on organisms, including hepatotoxicity [6], neurotoxicity [7], reproductive toxicity [8], developmental toxicity [9], immunotoxicity [10], thyroid disruption [11], cardiovascular toxicity [12], pulmonary toxicity [13], nephrotoxicity [14], and carcinogenicity [15]. PFAS are widely present in various media such as water [16], sediments [17], and soil [18], and they can persist in the environment for long periods through sediments. In addition to atmospheric transport and degradation of precursors, atmospheric and oceanic transport of PFSAs themselves may also significantly contribute to their long-range transport [19].

Human exposure to PFAS occurs mainly through diet, inhalation, and dermal contact, with dietary intake being the predominant pathway, and aquatic products serving as a significant exposure vector. Due to the widespread presence of PFAS in the environment, humans are inevitably exposed to these substances. Studies show that the risk of PFAS exposure is much higher for individuals in special environments and related industries than for the general population [20]. Inhalation, dermal contact, and oral ingestion are the main routes of human exposure to PFAS, with oral ingestion being the predominant route [21]. Water and food are the primary carriers for oral exposure [22]. Aquatic products, including fish and shellfish, are the main food types for oral exposure [22,23]. In addition to external exposure studies, there have been internal exposure indicators measuring PFAS concentrations in human serum and urine [24], as well as studies on PFAS concentrations between mothers and infants [25].

Human exposure to PFAS occurs mainly through diet, inhalation, and dermal contact. Among these, dietary intake is the predominant pathway, with aquatic products serving as a significant exposure vector. Due to the widespread presence of PFAS in the environment, humans are inevitably exposed to these substances. Studies show that individuals in special environments and related industries face a much higher risk of PFAS exposure compared to the general population [20]. The main routes of human exposure to PFAS include inhalation, dermal contact, and oral ingestion, with oral ingestion being the most important [21]. Water and food are the primary carriers of oral exposure [22]. Aquatic products, such as fish and shellfish, represent major food types contributing to oral exposure [22,23].In addition to external exposure studies, internal exposure indicators have been developed, including measurements of PFAS concentrations in human serum and urine [24]. There have also been studies comparing PFAS concentrations between mothers and infants [25].

EFSA (European Food Safety Authority), based on the effects of PFAS on the immune system, in 2020 adopted a physiologically based pharmacokinetic (PBPK) model, using a daily intake of 0.64 ng/kg bw as a starting point, identified perfluorooctanoic acid (PFOA), PFOS, perfluorononanoic acid (PFNA), and perfluorohexane sulfonic acid (PFHxS) as the primary PFAS monomers of concern, and established a tolerable weekly intake (TWI) recommendation of 4.4 ng/kg bw per week for the sum of the intakes of PFOA, PFNA, PFHxS, and PFOS [26]. EFSA estimated that exposure to these four PFAS accounts for about half of the lower bound exposure to all PFAS, with the remaining exposure mainly coming from heptafluorobutyric acid (PFBA) and perfluorohexanoic acid (PFHxA), but these two PFAS have shorter half-lives and were therefore not included in this metric [27]. In Switzerland, the sum of PFOS, PFOA, PFNA, and PFHxS in crustaceans and mollusks must not exceed 5.0 μg/kg [28]. In accordance with Commission Regulation (EU) 2023/915, which sets maximum levels for certain contaminants in foodstuffs, the sum of concentrations of PFOS, PFOA, PFNA, and PFHxS in crustaceans and bivalve molluscs is regulated at 5.0 μg/kg (wet weight) [29].

Focusing on Zhejiang Province as a typical coastal and industrial region, this study investigates the contamination characteristics, source apportionment, and health risks of PFAS in locally consumed aquatic products. As one of the major fluorochemical-producing areas in southeastern China with high per capita consumption of seafood, Zhejiang offers a critical context for examining PFAS pollution resulting from both industrial and dietary pathways [30]. While previous studies have reported PFAS in Eastern Chinese surface waters [31] and soils [32], little is known about PFAS levels in aquatic products—particularly locally prevalent shellfish and crustaceans—or the associated health risks for residents. Furthermore, although source apportionment methods such as USEPA’s UNMIX Model [33], PMF Model [34], and PCA-MLR Model [35] have been applied elsewhere, their application in Zhejiang may reveal unique regional sources and profiles. By integrating contaminant measurement with advanced source analysis and exposure assessment, this study aims to provide novel insights into the sources, distribution, and human health implications of PFAS in a Chinese industrial coast, addressing a significant gap in the current literature and enabling targeted risk management.

## 2. Results

### 2.1. Pollutant Characteristics

PFAS contamination is widespread in aquatic organisms. Among the 21 PFAS analyzed, 87.9% of the 306 shellfish and crustacean samples tested positive, though contamination levels varied significantly across groups. Detection rates followed this order: marine crustaceans (97.1%) > bivalves (88.8%) > freshwater crustaceans (76.1%). Samples with ≥10 PFAS monomers were rare but most frequent in bivalves (7.7%) and marine crustaceans (7.3%), compared to freshwater crustaceans (3.0%). Σ_21_PFAS concentrations varied widely (<LOD to 173 ng/g ww), with a median of 4.11 ng/g ww (95th percentile: 11.78 ng/g ww). Marine crustaceans had the highest median contamination (1.51 ng/g ww), followed by freshwater crustaceans (0.81 ng/g ww) and bi-valves (0.39 ng/g ww).

**Figure 1:**
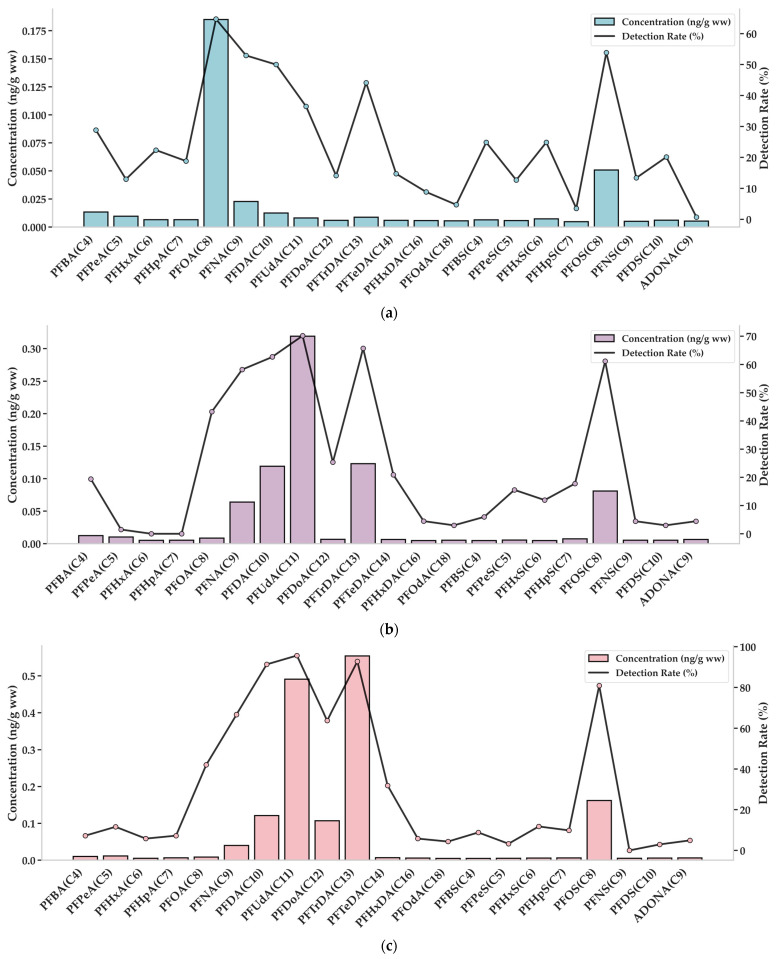
Graph of detection rates (%) of each pollutant and P_50_ concentration (ng/g ww). (**a**) Bivalves (shellfish), (**b**) freshwater crustaceans, and (**c**) marine crustaceans (abbreviations are provided in Table S1).

In bivalves, PFOA was the most frequently detected PFAS (64.7%), followed by PFOS (53.8%), PFNA (52.9%), PFDA (50.0%), and PFTrDA (44.1%). PFOA also showed the highest median concentration (0.1850 ng/g ww), exceeding PFOS (0.0508 ng/g ww), PFNA (0.0228 ng/g ww), and PFBA (0.0133 ng/g ww) (Figure 1a), indicating its dominant role in bivalve contamination. The co-occurrence of long- and short-chain PFAS suggests mixed pollution sources, including legacy compounds and emerging alternatives.

Among crustacean products, freshwater crustaceans showed the highest detection rate for PFUdA (70.1%), followed by PFTrDA (65.7%), PFDA (62.7%), and PFOS (61.2%), with PFUdA exhibiting the highest median concentration (0.3190 ng/g ww) (Figure 1b). In marine crustaceans, PFUdA was detected most frequently (95.7%), followed by PFTrDA (92.8%), PFDA (91.3%), and PFOS (80.9%), while the highest median concentration was recorded for PFTrDA (0.5540 ng/g ww) (Figure 1c). The overall contamination profile in crustaceans was dominated by long-chain PFAS, with PFUdA, PFTrDA, and PFDA being the most prevalent across both freshwater and marine species. The results indicate a distinct accumulation pattern of longer-chain compounds in crustaceans compared to bivalves.

Compliance with EU regulatory limits was assessed for PFOS, PFOA, PFNA, and PFHxS in shellfish and crustaceans (*n* = 306). The proportions of samples exceeding maximum levels (MLs) set by Regulations (EU) 2023/915 [29] were as follows: PFOS: 7/306 (2.3%; ML: 3.0 ng/g); PFOA: 66/306 (21.6%; ML: 0.7 ng/g); PFNA: 6/306 (2.0%; ML: 1.0 ng/g); PFHxS: 0/306 (ML: 1.5 ng/g); Σ_4_PFAS (sum of PFOS, PFOA, PFNA, and PFHxS): 15/306 (4.9%; ML: 5.0 ng/g). Exceedances were primarily observed in bivalves (57/66 PFOA-overlimit cases).

**Figure 2:**
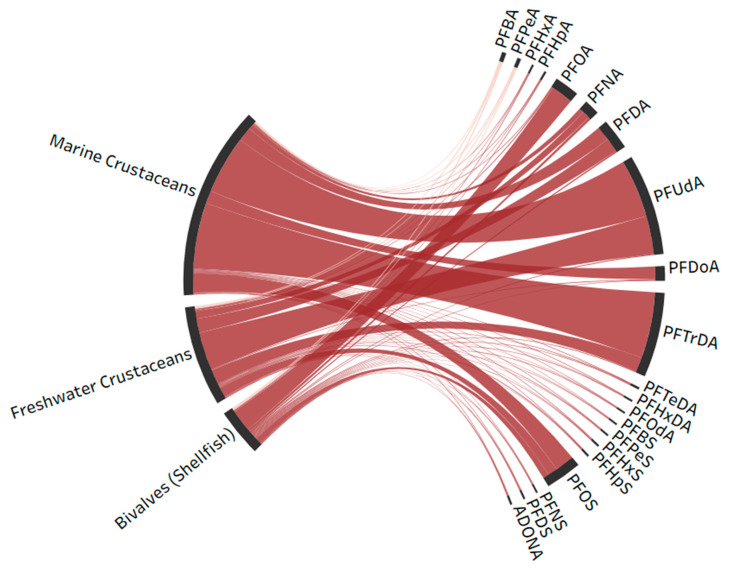
Sankey diagram of individual PFAS congeners’ concentrations across species. Link width and color intensity represent the magnitude of loading values for each congener within the species.

The Sankey diagram (Figure 2) revealed distinct PFAS distribution patterns among biological groups. Freshwater crustaceans were dominated by PFUdA (39.4%, C11), with significant contributions from PFTrDA (15.2%, C13) and PFDA (14.7%, C10), demonstrating preferential accumulation of odd-numbered long-chain PFCAs. Marine crustaceans showed stronger PFTrDA (35.3%, C13) loading than freshwater counterparts, while maintaining high PFUdA (31.3%, C11) levels. In contrast, bivalves exhibited unique PFOA (47.8%, C8) predominance with secondary PFOSs (13.1%).

### 2.2. Source Apportionment of 21 PFAS in Shellfish and Crustacean Samples

Prior to source apportionment, it should be noted that PFAS accumulation may vary across species; therefore, data from different types of organisms were not pooled directly. To assess differences in PFAS composition and concentration among bivalves, freshwater crustaceans, and marine crustaceans, this study applied PERMANOVA. The results revealed statistically significant differences among the three groups (F = 3.49, *p* < 0.001). Pairwise PERMANOVA tests were further conducted to identify specific differences between taxonomic categories. These tests indicated no significant difference between freshwater and marine crustaceans (F = 1.21, *p* = 0.2097), whereas significant differences were observed between bivalves and freshwater crustaceans (F = 5.54, *p* < 0.001) and between bivalves and marine crustaceans (F = 3.28, *p* < 0.001) (Text S1). Consequently, in subsequent source apportionment analyses using the EPA PMF 5.0 model, freshwater and marine crustaceans were combined into a single group.

The EPA PMF 5.0 model was employed to identify potential sources of PFAS contamination in bivalves. Following the official user guide, to ensure analytical accuracy, only contaminants with detection frequencies greater than 20% were included. Based on prior knowledge, three probable contamination sources were hypothesized: water pollution, industrial chemical release, and atmospheric deposition. Accordingly, the model was run with factor numbers ranging from 3 to 7, with each factor representing a potential pollution source. The optimal number of factors was determined based on the Q-value; too few factors may lead to incomplete separation of sources, while too many may indicate overfitting. In the results for bivalves, it was observed that when transitioning from the 4-factor model to the 5-factor model, both Q (Robust) and Q (True) decreased, while the Q (Robust)/Q (True) ratio increased. However, in the 6-factor model, the decreases in Q(Robust) and Q(True) were less pronounced, and the Q (Robust)/Q (True) ratio began to approach 1. Based on these findings, the 5-factor model was selected (Figure S1). After performing 200 bootstrap (BS) samplings, Factor 1 and Factor 4 exhibited poor stability (below 70%), while the remaining factors demonstrated stability approaching 90% (Figure S2). In the results for crustaceans, both Q (Robust) and Q (True) decreased significantly when transitioning from the 3-factor to the 4-factor model, accompanied by an increase in the Q (Robust)/Q (True) ratio. Subsequent increases in the number of factors resulted in less substantial reductions in Q (Robust) and Q (True) (Figure S3). Consequently, the 4-factor model was adopted. After conducting 100 bootstrap (BS) samplings, all factors except Factor 1 exhibited stability exceeding 80% (Figure S4). Ultimately, a four-factor solution was selected for crustaceans and a five-factor solution for bivalves.

#### 2.2.1. Crustacean Factor 1 and Factor 3: Sources from Different Industrial Processes (Figure 3a,c)

The primary contributing pollutants for Factor 1 were perfluorotetradecanoic acid(PFTeDA)(60.0%) and perfluorododecanoic acid(PFDoA) (20.7%). The predominant pollutants for Factor 3 were PFTrDA (45.3%) and PFUdA (41.1%). The major contributing pollutants in both Factor 1 and Factor 3 were long-chain PFCAs. Existing research indicates that long-chain PFCA production primarily involves three major chemical engineering processes. Using fluorotelomer olefins as starting compounds, oxidation predominantly yields odd-numbered PFCAs [36]. The oxidation process of fluorotelomer iodides mainly produces even-numbered PFCAs [37]. Studies on the textile industry revealed higher concentrations of perfluoroheptanoic acid(PFHpA), PFHxA, PFNA, PFDA, PFUdA, and PFTrDA in chemical fibers compared to cotton and other materials [38], suggesting that Factor 3 may be associated with the textile industry. Furthermore, alterations in light exposure and heterogeneity on particle surfaces (e.g., ash and mineral dust) may cause shifts in the odd-even PFCAs homologue ratio resulting from Fluorotelomer Alcohol (FTOH) oxidation [39].

**Figure 3:**
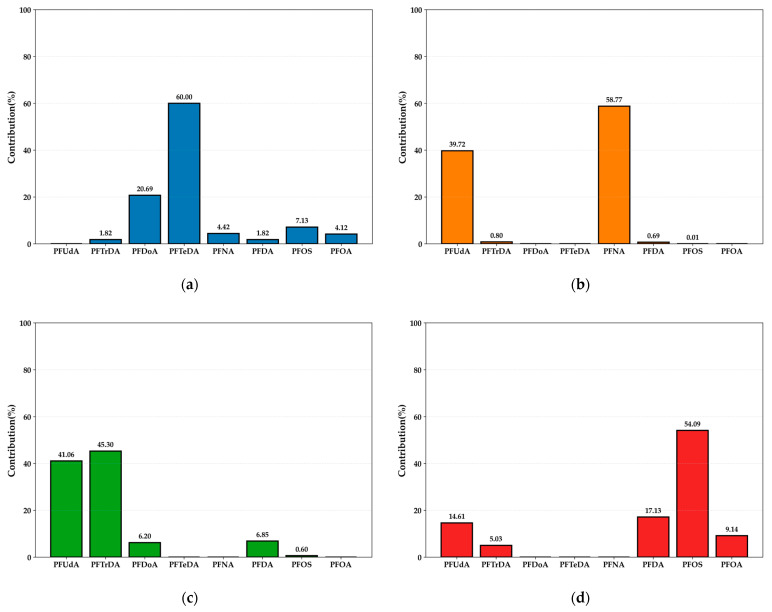
Proportion of individual PFAS compounds in crustaceans across different factors. (**a**) Factor 1; (**b**) Factor 2; (**c**) Factor 3; (**d**) Factor 4.

#### 2.2.2. Crustacean Factor 2: Domestic Waste Area Composite Pollution (Figure 3b)

Factor 2 primarily contributed to PFNA (58.8%) and PFUdA (39.7%) as pollutants. PFNA is commonly used in oil-proof coatings for textiles (outdoor apparel and carpets) and food packaging. PFNA may correlate with municipal solid waste leachate, with concentrations detected at 11–87 ng/L across three different leachate sites in Tianjin, China [40]. Similarly elevated PFNA concentrations (33.0–782.0 ng/L) were identified in leachate from the Toronto Hamilton Landfill in Canada [40]. PFUdA may also associate with the textile industry [38]. This factor is classified as a mixed factor related to domestic waste.

#### 2.2.3. Bivalves Factor 1: Short-Chain Fluorochemical Alternatives (Figure 4a)

Factor 1 primarily contributes pollutants as PFBA (88.4%) and PFOA (9.2%). PFBA serves as a short-chain alternative to PFOA [41]. Existing studies indicate that short-carbon-chain perfluorobutanesulfonic acid (PFBS) and PFBA are becoming dominant types in China’s environmental media [42]. Research on the Rhine River Basin demonstrates that wastewater from industrial treatment plants significantly increases PFBA and PFBS levels in downstream waters [43]. Investigations of two fluorochemical industrial parks in northern China’s Daling River Basin reveal electrochemical fluorination (ECF) processes as sources of linear and branched isomer mixtures of PFBS, PFBA, and PFOA [44]. This factor represents short-chain alternatives in traditional fluorochemical manufacturing.

**Figure 4:**
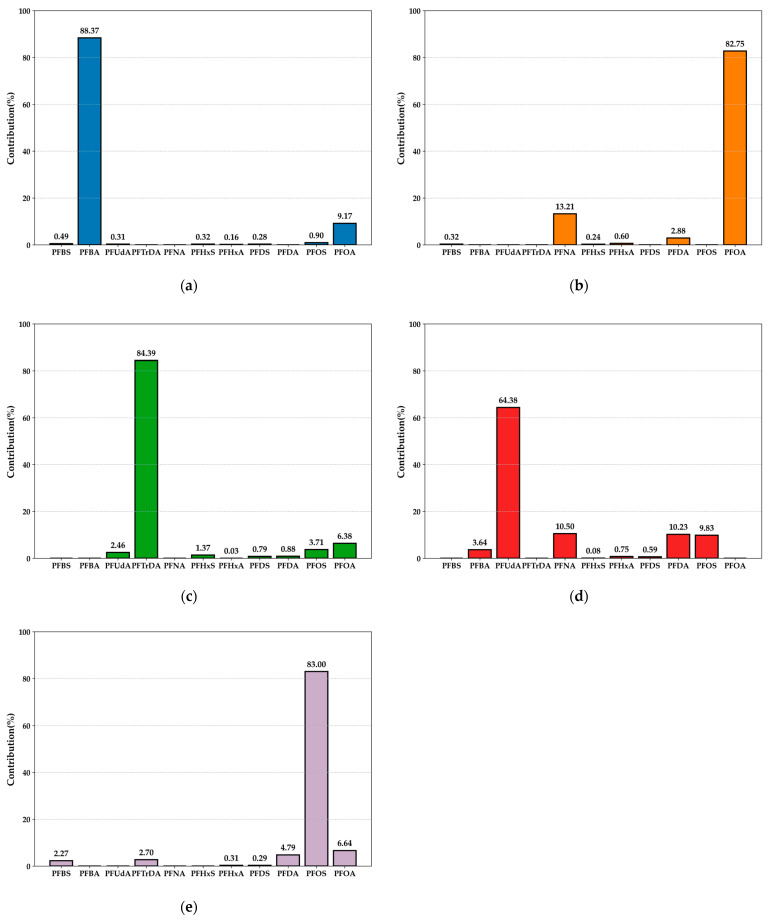
Proportion of individual PFAS compounds in bivalves across different factors. (**a**) Factor 1; (**b**) Factor 2; (**c**) Factor 3; (**d**) Factor 4; (**e**) Factor 5.

#### 2.2.4. Bivalve Factor 2: PTFE and PVDF Chemical Emissions (Figure 4b)

Factor 2 was primarily composed of PFOA (82.75%) and PFNA (13.21%). The Jiangsu Suzhou New District Fluorochemical Industrial Park, one of the largest fluorochemical bases in Asia, hosts over 20 major international fluorochemical companies. Its primary products, polytetrafluoroethylene (PTFE) and polyvinylidene difluoride (PVDF), are significant sources of PFOA and PFNA emissions into the environment [45]. The pollutant profile of this factor aligns closely with emissions from this industrial park, thus identifying this source as releases from PTFE and PVDF chemical production.

#### 2.2.5. Factor 3 and Factor 4 in Bivalves: Atmospheric Deposition from Specific Industrial Production (Figure 4c,d)

Factor 3 was predominantly contributed by PFTrDA (84.39%), while Factor 4 was primarily characterized by PFUdA (64.38%), PFDA (10.23%), and PFOS (9.83%). Both PFTrDA and PFUdA are long-chain odd-carbon-number PFCAs. Long-chain odd-carbon-number PFCAs are primarily associated with dust emissions from specific industrial processes [46]. Studies have shown that industrially emitted PFUnDA and PFTrDA ultimately persist in the environment within soil and dust. In a sampling study of soil and atmospheric dust around a PVDF and fluororubber production base near Lyon, France, PFUdA was identified as the predominant PFAS in surface soil (concentration range: 12–245 ng/g dw), whereas PFTrDA was more frequently detected in outdoor dust (concentration range: <0.5–59 ng/g dw [47]). These findings support the contribution of fluoropolymer manufacturing plants to the presence of long-chain odd-carbon-number PFCAs in the environment. Therefore, Factor 3 and Factor 4 in bivalves were categorized as atmospheric deposition from specific industrial production.

#### 2.2.6. Crustacean Factor 4 and Bivalve Factor 5: Historical PFOS Chemical Industry and Aqueous Film-Forming Foam (AFFF) Use (Figure 3d and Figure 4e)

In crustacean Factor 4, the main loadings were PFOS (54.1%), PFDA (17.1%), and PFUdA (14.6%). In bivalve Factor 5, the main loading was PFOS (83.0%). As surfactants and high-performance chemicals, PFOS compounds have been widely used in various consumer goods and industrial processes, such as firefighting foams, carpets, leather, fabrics, paper, cleaning products, pesticides, hydraulic fluids, semiconductors, photolithography, and metal plating [48]. A 2010 survey on annual PFOS emissions and source patterns at China’s provincial level revealed that textile treatment and metal plating industries were the primary PFOS sources in Zhejiang Province [49]. PFSAs like PFOS and PFHxS were key components in historical 3M electrochemical fluorination (ECF)-based aqueous film-forming foam (AFFF) formulations. Short-chain PFSAs (PFEtS and PFPrS), long-chain PFSAs (perfluorononanesulfonate(PFNS) and PFDS), and long-chain perfluorocarboxylic acids (PFCAs) (PFNA, PFDA, PFUdA, PFDoA, and PFTrDA) were also detected in ECF-based AFFF [50]. These two factors were thus identified as indicative of historical PFOS industrial and AFFF usage.

### 2.3. Exposure Levels

This study utilized P50 concentrations in crustacean and bivalve samples to assess the tolerable duration of exposure to Σ_4_PFAS (sum of PFOS, PFOA, PFNA, PFDA) and Σ_21_ PFAS via crustacean and bivalve consumption by adult consumers in Zhejiang Province.

Among 7354 adults, the mean Σ_4_PFAS estimated daily intake (EDI) was 0.050 ng/kg bw/day, with higher exposure in females (0.054) than males (0.046). The P95 high-consumption group reached 0.20 ng/kg bw/day, still below EFSA’s safety threshold (4.4 ng/kg_bw/week). Age-stratified analysis revealed significantly higher EDIs in younger adults (18–30 years: 0.060 ng/kg bw/day) versus adults ≥60 years (0.042 ng/kg bw/day) (*p* < 0.05 for pairwise comparison, Kruskal–Wallis/Dunn tests), showing a clear declining trend with age. (Table 1).

Among 7354 general adult individuals, the average Estimated Daily Intake (EDI) of Σ_4_PFAS was 0.050 ng/kg bw per day, with males averaging 0.046 ng/kg bw per day and females 0.054 ng/kg bw per day (Table 1). The exposure level in the high-consumption group (P_95)_ was 0.20 ng/kg bw per day. Both the general adult population and high-consumption group had EDIs below EFSA’s health-based guidance value of 0.63 ng/kg bw per day, indicating low health risks from Σ_4_PFAS exposure through crustaceans and bivalves in the study population. By age group, the highest average Σ_4_PFAS EDI occurred in the 18–30 years group (0.060 ng/kg bw per day), followed by 31–50 years (0.051 ng/kg bw per day), 51–59 years (0.047 ng/kg bw per day), and ≥60 years (0.042 ng/kg bw per day). EDI averages showed a decreasing trend with increasing age. Homogeneity of variance (Levene), Kruskal–Wallis H, and post hoc Dunn tests revealed significant differences in exposure levels across age groups (*p* < 0.05).

Among 7354 adult consumers, the estimated daily intake (EDI) of Σ_21_ PFAS ranged from 0.01 to 15.7 ng/kg bw/day, with a mean of 0.30 ng/kg bw/day. The high-consumption group (P95) exhibited an EDI of 1.22 ng/kg bw/day, four times higher than the general population (Table 2). In the general consumer group, the highest-exposure compounds were PFUdA, followed by PFTrDA, PFOS, PFDA, and PFDoA, while the remaining 16 PFAS showed minimal exposure levels. Notably, PFUdA and PFTrDA were the predominant exposure sources in the high-consumption group, with significantly higher levels than other compounds. Although PFOS and PFDA had relatively lower overall exposure, their elevated levels in high consumers (P95) warrant attention. These findings highlight the substantial influence of consumption habits on PFAS exposure and demonstrate that mean PFOS exposure was lower than that of PFUdA and PFTrDA in this population.

### 2.4. Health Risk Assessment

Based on the health guidance value for Σ_4_PFAS (sum of PFOS, PFOA, PFNA, PFDA) recommended by EFSA at 4.4 ng/kg bw per week, the health risks of Σ_4_PFAS exposure were evaluated across different age and gender groups. Among 7354 adult consumers, a small subset of consumers (0.31%, 23/7354) exhibited Hazard Quotient (HQ) values > 1, indicating potential health concerns. This subgroup was predominantly female (69.6%). Further analysis indicated substantial differences in dietary patterns between this exceeding group and the healthy population. Both average single-consumption quantity and annual consumption frequency in this exceeding group exceeded the overall population averages. Additionally, females’ relatively lower body weight combined with high consumption frequency and large single-serving intake significantly contributed to elevated Σ_4_PFAS exposure in this subgroup.

## 3. Discussion

Given the widespread presence of PFAS in the environment and the prominence of food contamination issues, this study focuses on shellfish and crustacean products in Zhejiang Province, systematically analyzing contamination characteristics of 21 PFAS and assessing exposure risks for adult consumers. Contamination characterization results indicate that PFAS contamination is prevalent in commercially available shellfish and crustaceans, and distinct interspecific variations are observed in the bioaccumulation of PFAS. Both marine and freshwater crustaceans demonstrated strong bioaccumulation capacity for long-chain PFCAs, PFUdA, and PFTrDA. In contrast, bivalves primarily accumulated PFOA and PFOS.

These differences may be attributed to the following biological characteristics: (1) Species-specific differences and metabolic capacity: Crustaceans (e.g., shrimp and crabs), as higher-trophic-level predators with higher lipid content, tend to accumulate long-chain PFCAs (e.g., PFUdA and PFTrDA) due to their stronger hydrophobicity and environmental persistence [51,52]. In contrast, filter-feeding bivalves primarily absorb PFAS passively from water, resulting in accumulation patterns more dependent on the concentrations of PFOA and PFOS in the aquatic environment [53,54]. (2) Trophic level and food chain position: Crustaceans generally occupy a higher trophic position than bivalves, potentially amplifying long-chain PFAS accumulation through predation [51]. Meanwhile, the filter-feeding behavior of bivalves exposes them directly to dissolved PFAS, with shorter-chain compounds (e.g., PFOA) being more water-soluble and mobile [55]. (3) Habitat influence: The bioaccumulation patterns of PFAS in freshwater crustaceans may differ from those in marine species due to variations in environmental contamination levels, such as higher industrial discharge leading to greater long-chain PFCAs accumulation in freshwater systems [56].

Both marine and freshwater crustaceans exhibited a strong capacity for bioaccumulation of long-chain perfluoroalkyl carboxylic acids (PFCAs), especially PFUdA and PFTrDA. This accumulation pattern can be attributed to several interrelated environmental and ecological factors. Aquatic habitats play a crucial role in shaping PFAS exposure profiles. Studies show that long-chain PFAS tend to accumulate more readily in sediment-rich environments through hydrophobic and electrostatic interactions [57]. Consequently, the ratio of long-chain to short-chain PFCAs increases significantly with distance from pollution sources [58]. As benthic organisms, many crustaceans live in close contact with sediments, leading to heightened exposure to long-chain compounds. Furthermore, the bioaccumulation potential of long-chain PFCAs and PFOS is amplified through trophic transfer. These substances demonstrate significant biomagnification within aquatic food webs [59], resulting in higher concentrations in organisms occupying elevated trophic levels, such as crustaceans. For instance, trophic magnification of PFUdA, PFDoA, and PFOS has been documented in subtropical food chains [60]. Notably, long-chain PFCAs (with carbon chain lengths > 10), particularly PFUdA, exhibit pronounced bioaccumulation tendencies [61]. In summary, the interplay between aquatic environmental conditions, sediment interaction, and food web dynamics collectively enhances the accumulation of long-chain PFCAs in crustaceans.

PFOA constitutes the primary PFAS contaminant in bivalve mollusk products from Zhejiang Province, with notably higher exceedance rates of EU Regulation 2023/915 [29] limits (21.6% of samples, predominantly in bivalve mollusk products) compared to PFOS (2.3%) and PFNA (2.0%). This pattern aligns with broader regional observations: a 2023 Shandong Province study found PFOA concentrations consistently exceeding PFOS homologues in shellfish [62], while a six-coastal-region survey across China reported PFOA accounted for 26–72% of total PFAS contaminants in bivalves [63]. The predominance of PFOA in bivalve mollusks can be attributed to three interrelated factors. First, bivalves’ filter-feeding physiology demonstrates greater efficiency at accumulating short-chain PFCAs like PFOA (C8) compared to long-chain PFSAs such as PFOS, due to fundamental differences in uptake and depuration kinetics [54,58]. Second, the historical widespread use of PFOA has created persistent environmental reservoirs, particularly in benthic ecosystems where many bivalve species reside, continuing to contaminate marine food webs despite recent regulatory restrictions. Third, their predominant habitat in brackish, shallow waters with sandy/muddy substrates facilitates direct bioaccumulation of contaminants near pollution sources or through riverine sewage discharge [64].

In this study, source apportionment of PFAS contamination in bivalves and crustaceans indicates that pollutants likely originate from multiple contamination pathways, including but not limited to landfill leachate, industrial wastewater discharge, the use of aqueous film-forming foams (AFFFs), and atmospheric deposition. These pathways contribute to the presence of PFAS in aquatic environments, leading to subsequent bioaccumulation in benthic and filter-feeding organisms. Studies indicate that perfluorooctanoic acid (PFOA) in agricultural biosolids can be redistributed via rainfall [65]. A Positive Matrix Factorization (PMF) model analysis of PFAS data from functional zones in Tianjin, conducted in 2022, identified major sources including textile processing, metal electroplating plants, and food packaging/coating materials [66]. Furthermore, a 2023 PMF analysis of serum PFAS levels in two U.S. communities discerned multiple exposure sources, such as AFFF-contaminated drinking water and consumer products [67]. Overall, these dominant pollution sources are consistent with the findings of this study, which primarily attribute contamination to domestic waste and industrial production.

This study reveals generally low Σ_4_PFAS (PFOA, PFOS, PFNA, and PFHxS) exposure levels among Zhejiang adults consuming bivalves and crustaceans, with minimal exceedance of EFSA’s health-based guidance value (Σ_4_PFAS), indicating controllable health risks. However, attention is warranted for higher-exposure monomers (PFUdA and PFTrDA) and secondary monomers (PFOS and PFDA). An internal PFAS exposure study documented temporal increases in long-chain PFCAs concentrations in marine organisms and human serum [68]. Zhoushan research demonstrated PFTrDA’s placental transfer (comparable maternal/cord blood concentrations) [69]. A Japanese birth cohort confirmed significant negative correlations between prenatal PFNA, PFDA, and PFTrDA exposure and infant birth weight/length (female infants showing greater PFTrDA sensitivity) [70]. The PFAS monomer exposure patterns herein align with internal exposure trends. Given relatively high PFUdA, PFTrDA, PFDA, and PFNA concentrations in bivalves and crustaceans, sensitive populations (e.g., pregnant women and children) should reduce consumption frequency during gestation to mitigate developmental toxicity risks.

This study has several limitations. Firstly, we adopted the P50 concentration to describe the overall PFAS profile in freshwater and marine crustaceans and as an indicator for calculating population exposure. This approach may underestimate extreme exposure scenarios. Random number imputation was used to handle non-detected values, aiming to reflect potential natural background contamination of PFAS. Such data processing methods may introduce a certain degree of uncertainty. The food frequency method employed in the 2015–2016 survey data may introduce recall bias, thereby affecting the results to some extent. Second, a potential limitation arises from the temporal gap between the dietary intake survey (conducted in 2015–2016) and the PFAS concentration measurements (obtained in 2023–2024). Shifts in dietary habits or contamination profiles over this period could introduce uncertainty to the exposure assessment. However, the 2015–2016 survey remains the most comprehensive and representative dataset available for aquatic product consumption in the study population. Moreover, consumption patterns of aquatic products in Zhejiang—a coastal region with established dietary traditions—are generally stable over time. Nonetheless, this chronological discrepancy is acknowledged as a limitation, and the need for future studies with temporally aligned data collection is emphasized in the Discussion. Furthermore, since the freshwater and marine crustacean samples commercially available in Zhejiang Province were sourced through distribution channels with often mixed origin information, this complicates source apportionment of PFAS in the samples and increases uncertainty.

## 4. Materials and Methods

### 4.1. Sample Collection

From 2023 to 2024, 306 samples of freshwater crustaceans, marine crustaceans, and bivalves were collected from representative and typical seafood markets in Zhejiang province by trained investigators, including 170 samples of bivalves, 69 samples of marine crustaceans, and 67 samples of freshwater crustaceans. After collection, the samples were transported to the laboratory via cold chain. Tested crustacean and shellfish species details with exact sample counts are provided in Table S2.

### 4.2. Sample Preparation and Analysis

A total of 21 PFAS compounds were determined using liquid chromatography-tandem mass spectrometry (LC-MS/MS), including 13 PFCAs (C4-C14, C16, and C18), 7 PFSAs (C4-C10), and one novel PFAS (ADONA). Isotope-labeled internal standards were employed for liquid chromatography, and the detection results were calculated based on the response values corrected by the internal standard method according to the standard calibration curve to determine concentrations (Mixed standard solutions, PFAC-MXC&PFAC-MXF, mass labeled PFAS mixed standard solutions MPFAC-C-ES (Wellington Laboratories, Guelph, ON, Canada). Controls, DIRM-NIP-PFAS-F (Wageningen University and Research, Wageningen, The Netherlands) (5ppb standard TIC plot Figure S5). In the spiked concentration range of 1–5 μg/kg, recovery rates were 80–110%, and under repeatability conditions, the absolute difference between two independent measurement results should not exceed 20% of the arithmetic mean.

For shellfish, the soft tissues are removed from the shell; for crustaceans, the edible muscle tissue is collected. The tissues are homogenized using a pre-cooled homogenizer (4 °C), with liquid nitrogen flash-freezing applied when necessary. Aliquots of ≥500 g of the homogenate are dispensed into acid-washed polypropylene centrifuge tubes. The tubes are labeled and stored in a −80 °C ultra-low temperature freezer. Blank control samples are collected simultaneously throughout the entire procedure. Sample testing must be completed within six months. A total of 2 g of the Shellfish and Crustaceans sample was placed in a 50 mL polypropylene tube and spiked with internal standards (2 ng isotopic labeling PFAS; Wellington Laboratories, ON, Canada). An amount of 8 mL acetonitrile was added after fully mixing using 2 mL of water in a Vortex oscillator (250 r/min) for 3 min, and extracted using ultrasonic oscillation for 30 min. The supernatant was collected after centrifugation. Finally, the extracts were purified using an HLB-P/HMR-Lipid solid-phase extraction column (Anavo, Beijing, China). Amounts of 1.5 g sodium chloride were added, and after fully mixing, 4 mL of supernatant was concentrated with nitrogen until nearly dry and then diluted with methanol to a constant volume of 0.2 mL. Quantification was performed by isotope dilution using an ultra-performance liquid chromatography/tandem mass spectrometry instrument (Xevo TQ-S; Waters, Milford, MA, USA). All reagents and consumables have been tested for blank to control background interference before use, and a spiked experiment has been added to each batch (10 samples) to ensure the accuracy of the experiment. Use DIRM-NIP-PFAS-F quality control samples for quality control in each experiment. The analytical method was optimized for the simultaneous determination of 21 PFAS using an Acquity UPLC^®^ system (Waters Corp., Milford, MA, USA) coupled to a mass spectrometer (MS) (Xevo TQ-S, Waters Corp.). Chromatographic separation was performed using a mobile phase containing 2 mM ammonium acetate in water and methanol, a flow rate of 0.3 mL/min, and an Acquity UPLC^®^HSS (Waters, Milford, MA, USA)T3 (1.8 µm, 2.1 mm × 100 mm, Waters Corp.) at a column temperature of 45 °C ± 0.5 °C. The MS was operated using a negative electrospray ionization interface. Multiple reaction monitoring transitions were used for quantification; m/z information and instrument conditions are shown in Table S3. The MRM transitions monitored for 21 PFAS are shown in Table S4. Data acquisition and analysis were performed using Masslynx 4.1 software (Waters Corp.). After pretreatment, most target compounds exhibited minimal to moderate matrix effects. To achieve more accurate quantification, isotope-labeled internal standards were applied for concentration correction. After correction, the relative recoveries of the target compounds fell within the range of 85% to 115%. Therefore, the reported results were not subjected to further recalculations based on recovery rates.

When the sample amount is 2 g and the constant volume is 10.0 milliliters, with the constant volume of the sample solution being 0.2 milliliters, the detection limits for PFBA and PFPeA are 0.02 μg/kg, and the quantification limit is 0.06 μg/kg. For the remaining 19 perfluorinated compounds, the detection limit is 0.01 μg/kg and the quantification limit is 0.03 μg/kg. Partial samples TIC diagrams are shown in Figures S6 and S7.

### 4.3. Consumption Data Collection

Shellfish and crustacean consumption data were derived from the food consumption survey conducted in Zhejiang Province between 2015 and 2016. The survey was carried out in 10 cities and 18 counties/county-level cities in Zhejiang Province, with survey site selection following the Probability Proportional to Size (PPS) sampling principle. Three townships (sub-districts) were selected in each county/city, two villages (neighborhood committees) were selected in each township (sub-district), and finally, 50 households were randomly selected in each village (neighborhood committee). A food frequency questionnaire was used to record the consumption status of participants. All participants signed informed consent forms, and their personal information was kept confidential. To ensure data quality, the project team provided unified technical training to all investigators before the survey, standardizing survey forms, interview procedures, and questionnaire completion standards. A three-level review mechanism was implemented for the questionnaires.

**Figure 5:**
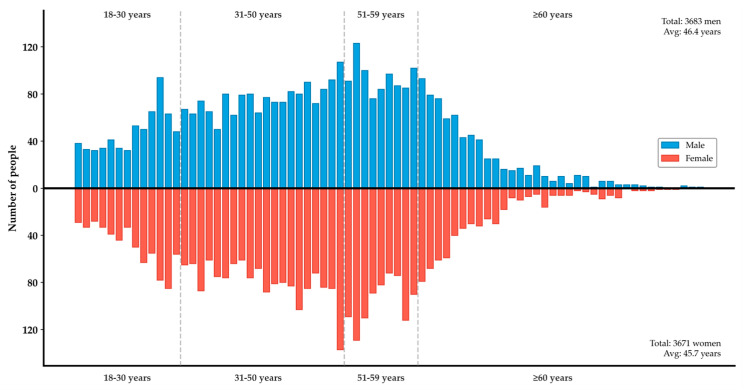
Survey participants’ gender and age distribution chart. Blue represents males, red represents females.

Approximately 19,968 residents aged 3 years and above completed the food frequency questionnaire. After the survey, the raw data were cleaned, excluding records with extreme outliers and missing key information. Ultimately, 7354 adults (aged ≥18 years) who had consumed at least one type of marine crustacean, freshwater crustacean, or bivalve were selected and included in this analysis. These participants were divided into four groups: 18–30 years, 31–50 years, 51–59 years, and ≥60 years (Figure 5).

### 4.4. Data Quality Control

Strict quality control measures were implemented throughout sampling, extraction, and instrumental analysis. Cold-chain transportation was maintained during sample collection and analysis, with transport blanks incorporated. Samples exhibiting abnormally high or low values underwent retesting (parallel analysis with original samples) to confirm authentic contamination. Outlier data verified as genuine contamination were retained after review, with enhanced data auditing and standardized documentation. Recovery rates of spiked samples were measured alongside detection limits (LOD), quantification limits (LOQ), and laboratory blanks. By spiking low-concentration samples, the spiked concentration when the signal-to-noise ratio (S/N) is 3 is defined as the limit of detection (LOD), while the concentration when S/N reaches 10 is defined as the limit of quantification (LOQ). The value of non-detected samples was assumed to be the LOD divided by 2 in the exposure assessment and risk characterization. The LOD and LOQ values for all analyzed samples are detailed in the supporting document (Table S5).

### 4.5. Data Analysis

Data analysis and visualization were performed using Python 3.12, R 4.43, and Excel 2019MSO (2304 Build 16.0.16327.20200). Normality of the data was assessed using the Shapiro–Wilk test. For data that followed a normal distribution and met the assumption of homogeneity of variance (as evaluated by Levene’s test), an independent samples *t*-test was applied for between-group comparisons. For data that did not follow a normal distribution, the Kruskal–Wallis H test was used to analyze intergroup differences. If the Kruskal–Wallis test indicated significant differences, post hoc pairwise comparisons were conducted using Dunn’s test. All statistical analyses were two-tailed, and a *p*-value < 0.05 was considered statistically significant.

### 4.6. Source Analysis of PFAS

Source analysis was employed using the EPA-recommended PMF 5.0 software. Given the PMF model’s sensitivity to zero values and potential zero-inflation issues, values below LOD were not simply substituted with zero. Due to potential background PFAS contamination, assigning LOD/2 to non-detects might obscure actual pollution signals. Therefore, values below LOD in the PMF model were reassigned as random numbers between 0 and LOD, while detections between LOD and LOQ were calculated as LOQ/2 [34]. Interspecies differences in contaminant profiles were evaluated using PERMANOVA. This method decomposed total variance through Euclidean distances to quantify how PFAS compositions explained differences among species groups, with permutation tests determining statistical significance.

The EPA-recommended PMF5.0 model was used for source apportionment of PFAS; this model defines the sample matrix as the product of two unknown factor matrices with a residual matrix:

(1) X = G F + E

The sample matrix (*X*) consists of n observed samples and m chemical substances, *F* is the chemical profile matrix of *p* factors or sources, the *G* matrix describes the contribution of each factor to any given sample, and *E* is the residual matrix [35]. Indicators with detection rates exceeding 20% in bivalves and crustaceans were screened for input into the PMF model for source apportionment. For uncertainty estimation in the PMF model, following EPA guidelines: values below the LOD were assigned an uncertainty of 5/6 LOD, while values above the LOD were assigned an uncertainty estimated as follows:

(2) $$\mathrm{Uncertainty}\;=\;\sqrt{{\left(\mathrm{error}\times \mathrm{concentration}\right)}^{2}+{\mathrm{LOD}}^{2}}$$

The error rate is estimated at 0.1 based on experimental experience. Concentration represents the detected value (ng/g ww), while LOD denotes the contaminant’s detection limit (ng/g ww). As the PMF model requires a complete matrix for source apportionment, missing values in non-detected samples were addressed by filling concentrations with medians and uncertainties with 4-fold median uncertainties [71]. The optimal factor number was determined through Q (Robust)/Q (True) and Q-value decline analysis, with model stability assessed via BS Mapping [72].

### 4.7. Chronic Exposure Assessment

This study employed a dietary exposure assessment model based on subjects’ actual body weights, collecting data on population-specific actual consumption amounts and consumption frequency through questionnaires. Given the highly right-skewed distribution of target contaminant concentrations (Shapiro–Wilk test, *p* < 0.001), the median contaminant concentration (P_50_) was used instead of the arithmetic mean for exposure calculations to mitigate the impact of extreme values on risk assessment. The PFAS exposure levels across different populations were evaluated by applying the following exposure calculation formula:

(3) $${\mathrm{EDI}}_{i\;}=\;\frac{C_{i}\times \;{\mathrm{IR}}_{i}\times \;{\mathrm{EF}}_{i}\times \mathrm{ED}}{\mathrm{BW}\times \mathrm{AT}}$$

*C_i_* denotes the P_50_ concentration of PFAS in different food (ng/g, ww), *IR_i_* represents an individual’s daily consumption of freshwater fish species i (g/day, ww), *EF_i_* is the exposure frequency (days/year), *ED* indicates the duration of exposure, *AT* signifies the averaging time for non-carcinogenic effects (365 days/year × ED), and BW denotes an individual’s actual body weight (kg) [73].

This study assessed exposure levels to 21 PFAS congeners and Σ_4_PFAS in the adult population, with stratified analyses conducted across age groups (18–30, 31–45, 46–60, >60 years) and gender.

Current internationally adopted PFAS exposure assessment metrics are presented in Table 3. For health risk characterization, this study utilized the European Food Safety Authority’s (EFSA) Σ_4_PFAS health-based guidance value (TWI = 4.4 ng/kg_bw/week) as the benchmark, evaluating risk levels through calculation of the hazard quotient (HQ):

(4) $$\mathrm{HQ}=\frac{\mathrm{EDI}\times 7}{\mathrm{TWI}}$$

An HQ value below 1 is considered an acceptable risk.

## 5. Conclusions

This study describes the detection and distribution characteristics of 21 PFAS in commercially available shellfish and crustacean products in Zhejiang, analyzes the sources of PFAS, assesses the long-term dietary exposure risk of Σ_4_PFAS in the adult population, and explores the levels of individual and combined PFAS exposure. PFAS were commonly detected in shellfish and crustacean samples, with bivalves frequently contaminated by PFOA, PFOS, PFNA, PFDA, and PFTrDA. For crustaceans—regardless of marine or freshwater origin—PFUdA, PFTrDA, PFDA, and PFOS showed higher detection frequencies. Both marine and freshwater crustaceans demonstrated enrichment capabilities for long-chain perfluorocarboxylic acids (PFCAs), particularly PFUdA and PFTrDA. In contrast, bivalves primarily accumulated perfluorooctanoic acid (PFOA) and perfluorooctane sulfonate (PFOS), suggesting divergent uptake mechanisms or exposure pathways.

Source analysis indicated that crustacean PFAS originate mainly from mixed pollution of chemical industrial discharges and domestic waste, while bivalve contamination relates to short-chain fluorochemical substitutes, industrial atmospheric deposition, and releases from PTFE/PVDF production. Both pathways are co-influenced by historical PFOS production and aqueous film-forming foam (AFFF) usage. Chronic dietary exposure assessment indicated low Σ_4_PFAS risk for adults consuming crustaceans and bivalves. However, PFUdA and PFTrDA were the primary exposure sources for both general and high-consumption populations. Furthermore, exposure to PFOS and PFDA among high consumers (P95) requires particular attention.

Despite these findings, important knowledge gaps remain regarding the long-term health effects of low-dose exposure to the other 16 PFAS detected at lower levels, as well as their potential combined toxicological effects through synergistic interactions. The widespread detection of PFAS in these seafood products underscores the need for continued monitoring. Future research should focus on three key areas: source apportionment to better identify major contamination pathways, temporal trend analysis to track changes in PFAS levels in relation to industrial activity and regulatory measures, and a comprehensive assessment of combined toxicity effects. This study highlights the critical importance of integrating environmental monitoring with human health risk assessments to develop effective regulatory policies and ensure food safety for consumers. Furthermore, special consideration should be given to vulnerable populations, such as pregnant women and children, to establish tailored protective measures that safeguard their health. The findings provide valuable baseline data for ongoing surveillance of PFAS contamination in marine food products and contribute to our understanding of exposure risks associated with seafood consumption.

## Figures and Tables

**Table 1:** Exposure levels of Σ_4_PFAS among adults of different age groups and genders.

		EDI (Σ_4_PFAS) ng/kg bw per Day					
Age Groups	N	P_5_	P_50_	Mean	Std	P_95_	Max
18–30	1243	0.0019	0.026	0.06	0.097	1.04	0.22
31–50	3109	0.0010	0.022	0.051	0.083	0.82	0.20
51–59	1712	0.00093	0.017	0.047	0.10	2.16	0.19
>=60	1290	0.00071	0.014	0.042	0.088	1.88	0.17
**Gender**							
Man	3683	0.0011	0.019	0.046	0.082	0.17	2.16
Woman	3671	0.0010	0.020	0.054	0.099	0.21	1.88
**Total**	7354	0.0011	0.019	0.05	0.091	0.20	2.16

**Table 2:** Exposure levels of 21 types of PFAS in the adult population.

Group	EDI (21 PFAS) (ng/kg bw per Day)					
	P_5_	P_50_	Mean	Std	P_95_	Max
PFBA	0.000065	0.0011	0.0028	0.0049	0.011	0.10
PFPeA	0.000061	0.0011	0.0027	0.0049	0.011	0.11
PFHxA	0.000029	0.00051	0.0013	0.0023	0.0050	0.050
PFHpA	0.000033	0.00058	0.0015	0.0027	0.0058	0.062
PFOA	0.000055	0.0016	0.0057	0.014	0.024	0.28
PFNA	0.00028	0.0047	0.012	0.021	0.046	0.43
PFDA	0.00055	0.011	0.028	0.051	0.11	1.21
**PFUdA**	**0.0016**	**0.036**	**0.098**	**0.19**	**0.39**	**4.91**
PFDoA	0.000062	0.0040	0.016	0.035	0.069	1.07
**PFTrDA**	**0.00086**	**0.027**	**0.090**	**0.19**	**0.38**	**5.54**
PFTeDA	0.000037	0.00064	0.0016	0.0029	0.0063	0.065
PFHxDA	0.000030	0.00052	0.00134	0.0024	0.0053	0.056
PFOdA	0.000029	0.00049	0.0012	0.0022	0.0047	0.045
PFBS	0.000027	0.00048	0.0012	0.0022	0.0047	0.047
PFPeS	0.000030	0.00052	0.0013	0.0023	0.0050	0.049
PFHxS	0.000030	0.00054	0.0014	0.0025	0.0054	0.057
PFHpS	0.000036	0.00063	0.0016	0.0028	0.0062	0.06
**PFOS**	**0.00055**	**0.011**	**0.031**	**0.060**	**0.13**	**1.62**
PFNS	0.000029	0.00051	0.0013	0.0023	0.0050	0.051
PFDS	0.000031	0.00055	0.0014	0.0025	0.0055	0.057
ADONA	0.000035	0.00061	0.0015	0.0028	0.0060	0.061
Σ_21_PFAS	0.0053	0.11	0.30	0.58	1.22	15.7

**Table 3:** Several PFAS exposure assessment indicators.

	Limit	Target PFAS	Year
EFSA recommended TWI	4.4 ng/kg_bw/week	Sum of exposure to PFOS, PFHxS, PFNA, and PFOA	2020 [26]
EPA estimated RfD	0.02 ng/kg bw/day	PFOA, PFOS	2021 [74]
The Department of Health, Food Standards Australia New Zealand (FSANZ)	22.85 ng/kg bw/day	PFOA	2017 [75]

## Data Availability

The data presented in this study are available upon request from the corresponding author due to restrictions, e.g., privacy or ethical reasons.

## Supplementary Materials

The following supporting information can be downloaded at: https://www.mdpi.com/article/10.3390/md23090359/s1, Table S1. Abbreviations; Table S2. Sample Categories and Specific Types; Table S3. MS instrument conditions; Table S4. MRM transitions monitored for each of the 21 PFAS congeners; Table S5. Pollutant Classification and Corresponding LOD and LOQ Values; Text S1. PERMANOVA Explanation; Figure S1. Q-value variation plot for bivalves, with blue representing Q(Robust), red representing Q(True), and a dashed line indicating Q(Robust)/Q(True); Figure S2. BS Mapping results for bivalves; Figure S3. Q-value variation plot for crustaceans, with blue representing Q(Robust), red representing Q(True), and a dashed line indicating Q(Robust)/Q(True); Figure S4. BS Mapping results for crustaceans; Figure S5. 5ppb standard TIC plot; Figure S6. A bivalve shellfish sample’s TIC diagram; Figure S7. A crustacean sample’s TIC diagram. [76,77,78] are references in the Supplementary Materials.
