# Supplementary material for: Contamination Characteristics of 21 PFAS in Shellfish and Crustaceans of Zhejiang Province and Exposure Risk Assessment for Adult Dietary Consumers
Source: Mar Drugs. 2025 Sep 15;23(9):359. doi: 10.3390/md23090359 (PMC12471464; doi:10.3390/md23090359)
Supplement: Supplementary file 1 [file marinedrugs-23-00359-s001.zip › marinedrugs-3870999-supplementary.pdf]

Table S1. Abbreviations

Full name	Abbreviations
Perfluoroalkyl and polyfluoroalkyl substances	PFASs
perfluoroalkyl carboxylic acids	PFCAs
Perfluoroalkyl Sulfonic Acids	PFSAs
Perfluorobutanoic acid	PFBA(C4)
Perfluoropentanoic acid	PFPeA(C5)
Perfluorohexanoic acid	PFHxA(C6)
Perfluoroheptanoic acid	PFHpA(C7)
Perfluorooctanoic acid	PFOA(C8)
Perfluorononanoic acid	PFNA(C9)
Perfluorodecanoic acid	PFDA(C10)
Perfluoroundecanoic acid	PFUdA(C11)
Perfluorododecanoic acid	PFDoA(C12)
Perfluorotridecanoic acid	PFTrDA(C13)
Perfluorotetradecanoic acid	PFTeDA(C14)
Perfluorohexadecanoic acid	PFHxDA(C16)
Perfluorooctadecanoic acid	PFOdA(C18)
Perfluorobutanesulfonic acid	PFBS(C4)
Perfluoropentanesulfonic acid	PFPeS(C5)
Perfluorohexanesulfonic acid	PFHxS(C6)
Perfluoroheptanesulfonic acid	PFHpS(C7)
Perfluorooctanesulfonic acid	PFOS(C8)
Perfluorononanesulfonic acid	PFNS(C9)
Perfluorodecanesulfonic acid	PFDS(C10)
4,8-Dioxa-3H-perfluorononanoic acid	ADONA(C9)
Tolerable weekly intake	TWI
Limit of Detection	LOD
Limit of Quantification	LOQ
Hazard Quotient	HQ
Estimated Daily Intake	EDI

Table S2. Sample Categories and Specific Types.

FOOD CATEGORY	Specific category	Latin Scientific Name	Sample Size
Bivalves			170
	Asiatic Hard Clam	Meretrix meretrix	1
	Baby Surf Clam	Mactra veneriformis	1
	Black Tiger Shrimp	Penaeus monodon	2
	Blood Ark Shell	Scapharca broughtonii	2
	Blood Cockle	Tegillarca granosa	1
	Blue Mussel	Mytilus edulis	1
	Chinese Mitten Crab	Eriocheir sinensis	1
	Chinese White Shrimp	Fenneropenaeus chinensis	8
	Cyclina Clam	Cyclina sinensis	4
	Freshwater Crayfish	Procambarus clarkii	31
	Freshwater Shrimp	Macrobrachium spp.	9
	Giant Tiger Prawn	Penaeus monodon	2
	Greasyback Shrimp	Metapenaeus ensis	3
	Hard Clam	Mercenaria mercenaria	5
	Mantis Shrimp	Oratosquilla oratoria	1
	Marine Shrimp	Penaeidae spp.	1
	Mud Crab	Scylla serrata	3
	Ninghai Razor Clam	Sinonovacula constricta	1
	Oriental River Prawn	Macrobrachium nipponense	21
	Pacific Oyster	Crassostrea gigas	7
	Pacific Whiteleg Shrimp	Litopenaeus vannamei	11
	Penaeid Shrimp	Penaeidae spp.	31
	Razor Clam	Solen spp.	10
	Red Shrimp	Solenocera spp.	2
	Red Swamp Crayfish	Procambarus clarkii	2
	Rough Shrimp	Trachysalambria curvirostris	3
	Short-necked Clam	Ruditapes philippinarum	1
	Shrimp (General Term)	Caridea	1
	Soft-shell Shrimp	Penaeus spp.	1
	Swimming Crab	Portunus trituberculatus	2
	Sword Prawn	Parapenaeus spp.	1
Freshwater crustaceans			67
	Asiatic Hard Clam	Meretrix meretrix	4
	Blood Cockle	Tegillarca granosa	3
	Blue Mussel	Mytilus edulis	9
	Edible Oyster	Ostrea edulis	2
	Hard Clam	Mercenaria mercenaria	2
	Pacific Oyster	Crassostrea gigas	19
	Razor Clam	Solen spp.	25
	Short-necked Clam	Ruditapes philippinarum	3
Marine crustaceans			69
	Blood Ark Shell	Scapharca broughtonii	2
	Blood Cockle	Tegillarca granosa	5
	Blue Mussel	Mytilus edulis	12
	Pacific Oyster	Crassostrea gigas	3
	Razor Clam	Solen spp.	17
	Red Swamp Crayfish	Procambarus clarkii	25
	Short-necked Clam	Ruditapes philippinarum	5
Total			306

Table S3.MS instrument conditions

Flow rate	0.3 mL/min
Injection Volume	3 μL
Column Temperature	45 ℃
Mobile Phase	2 mM ammonium format aqueous: methanol = 20:80→5:95 gradient
Temperature；Source	120 ℃
Desolvation	350 ℃
Gas Flow；Cone	Nitrogen, 150 L/Hr
Desolvation	Nitrogen, 650 L/Hr
Voltage; Cone	40-50 V
Capillary	1.5 kV
Collision	Argon, 10 eV
Ionization	ESI-Negative

Table S4. MRM transitions monitored for each of the 21 PFAS congeners.

compound	Precursor Ion（m/z）	Product Ion（m/z）	ISTD	Cone Voltage（V）	Collision Energy（eV）
PFBA	213.0	169.0*	13C4-PFBA	20	8
	213.0	69.0		20	10
PFPeA	262.9	218.9*	13C5-PFPeA	24	8
	262.9	69.0		24	10
PFHxA	312.9	268.9*	13C5-PFHxA	8	10
	312.9	118.9		8	16
PFHpA	362.9	318.9*	13C4-PFHpA	22	10
	362.9	168.9		22	14
PFOA	412.9	368.9*	13C8-PFOA	18	10
	412.9	168.9		18	18
PFNA	462.9	418.9*	13C9-PFNA	8	10
	462.9	218.9		8	14
PFDA	512.9	468.9*	13C6-PFDA	24	10
	512.9	218.9		24	181
PFUdA	562.9	518.9*	13C7-PFUdA	10	10
	562.9	268.9		10	18
PFDoA	612.9	568.9*	13C2-PFDoA	30	12
	612.9	168.9		30	22
PFTrDA	662.9	618.9*	13C2-PFTrDA	30	10

	662.9	168.9		30	26
PFTeDA	712.9	668.9*	13C2-PFTeDA	34	12
	712.9	168.9		34	28
PFHxDA	812.9	768.9*	13C2-PFTeDA	30	14
	812.9	168.9		30	32
PFODA	912.9	868.9*	13C2-PFTeDA	30	14
	912.9	268.9		30	30
L-PFBS	298.9	79.9*	13C3-PFBS	76	26
	298.9	98.8		76	24
L-PFHxS	398.9	79.9*	13C3-PFHxS	20	36
	398.9	98.8		20	30
L-PFOS	498.9	79.9*	13C8-PFOS	20	42
	498.9	98.8		20	38
L-PFDS	598.9	79.9*	13C7-PFUdA	20	52
	598.9	98.9		20	42
PFNS	548.5	79.8*	13C7-PFUdA	4	48
	548.5	98.9		4	44
ADONA	376.7	84.9*	13C4-PFHpA	2	30
	376.7	250.8		2	12
PFPeS	348.6	79.9*	13C5-PFHxA	20	28
	348.6	98.8		20	28
PFHpS	448.9	79.9*	13C8-PFOA	94	36
	448.9	98.8		94	32
13C5-PFHxA	317.7	272.8		214	8
13C4-PFBA	216.9	171.9		8	8
13C8-PFOA	420.6	375.7*		2	18
	420.6	171.8		2	10
13C9-PFNA	471.7	426.7*		2	10
	471.7	171.8		2	16
13C6-PFDA	518.6	473.7*		2	12
	518.6	222.8		2	18
13C7-PFUdA	569.6	524.7*		8	12
	569.6	273.8		8	18
13C2-PFDoA	614.9	569.9*		30	12
	614.9	169.1		30	26
13C3-PFHxS	401.6	79.8*		14	32
	401.6	98.7		14	34

13C8-PFOS	506.6	79.8*		2	44
	506.6	98.7		2	44
13C5-PFPeA	267.7	222.8*		14	8
13C3-PFBS	301.5	82.8*		34	24
	301.5	120.9		34	26
13C4-PFHpA	366.5	168.8		30	16
	366.5	321.8*		30	8
13C2-PFTrDA	664.8	169.1*		28	24
	664.8	319.3		28	18
13C2-PFTeDA	714.5	169.0		12	32
	714.5	669.6*		12	14

Table S5. Pollutant Classification and Corresponding LOD and LOQ Values

Category	LOD	LOQ
**PFCAs**		
Perfluorobutanoic acid (PFBA)	0.02	0.06
Perfluoropentanoic acid (PFPeA)	0.02	0.06
Perfluorohexanoic acid (PFHxA)	0.01	0.03
Perfluoroheptanoic acid (PFHpA)	0.01	0.03
Perfluorooctanoic acid (PFOA)	0.01	0.03
Perfluorononanoic acid (PFNA)	0.01	0.03
Perfluorodecanoic acid (PFDA)	0.01	0.03
Perfluoroundecanoic acid (PFUdA)	0.01	0.03
Perfluorododecanoic acid (PFDoA)	0.01	0.03
Perfluorotridecanoic acid (PFTrDA)	0.01	0.03
Perfluorotetradecanoic acid (PFTeDA)	0.01	0.03
Perfluorohexadecanoic acid (PFHxDA)	0.01	0.03
Perfluorooctadecanoic acid (PFOdA)	0.01	0.03
**PFSAs**		
Perfluorobutanesulfonic acid (PFBS)	0.01	0.03
Perfluoropentanesulfonic acid (PFPeS)	0.01	0.03
Perfluorohexane sulfonic acid (PFHxS)	0.01	0.03
Perfluoroheptanesulfonic acid (PFHpS)	0.01	0.03
Perfluorooctanesulfonic acid (PFOS)	0.01	0.03
Perfluorononanesulfonic acid (PFNS)	0.01	0.03
Perfluorodecanesulfonic acid (PFDS)	0.01	0.03
**New-PFASs**		
4,8-dioxa-3H-perfluorononanoic acid (ADONA)	0.01	0.03

Text S1. PERMANOVA Explanation

Nonparametric multivariate analysis of variance (PERMANOVA) is fundamentally an F-statistic-based approach that partitions total variance using distance matrices[1]. This method decomposes total variance through semi-metric (e.g., Bray-Curtis) or metric distance matrices (e.g., Euclidean) to quantify the explanatory power of different grouping factors or environmental variables on sample dissimilarity, with permutation tests evaluating the statistical significance of partitions[1]. Previous studies employed PERMANOVA to test significant differences in gross, dry, and ash-free heat of combustion (HoC) among fuel groups, and among fuel conditions, components, and size classes nested within major categories[2]. In multifactor PERMANOVA, grouping factors and environmental variables are treated as non-independent covariates that collectively influence community composition across groups. The analysis considers all factors as an integrated whole before partitioning variance to determine each factor's contribution to sample dissimilarity[3].

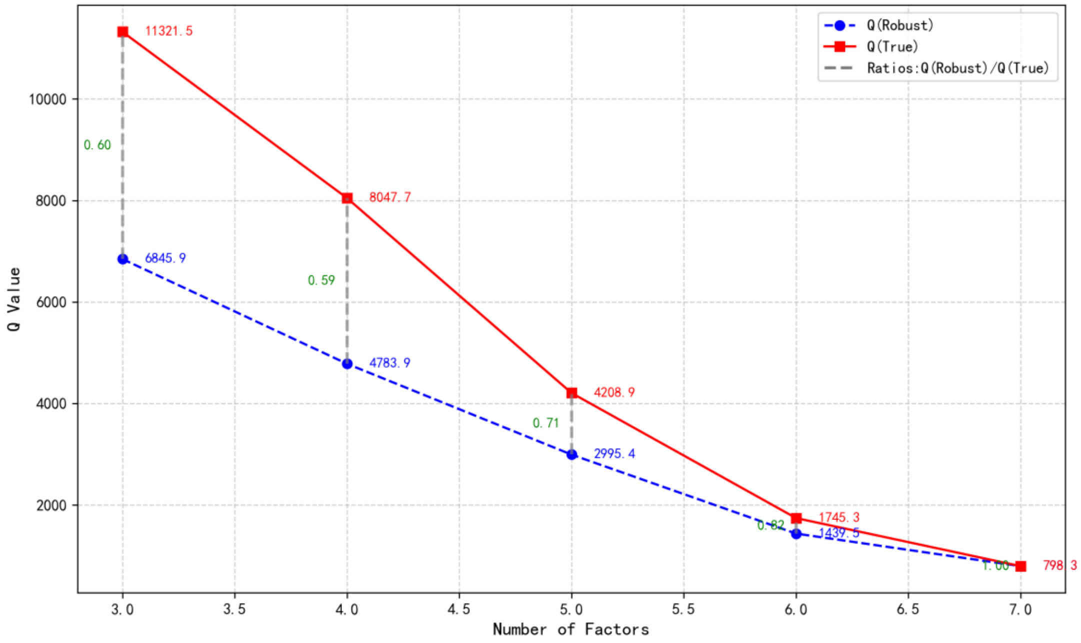

Figure S1. Q-value variation plot for bivalves, with blue representing Q(Robust), red representing Q(True), and dashed line indicating Q(Robust)/Q(True).

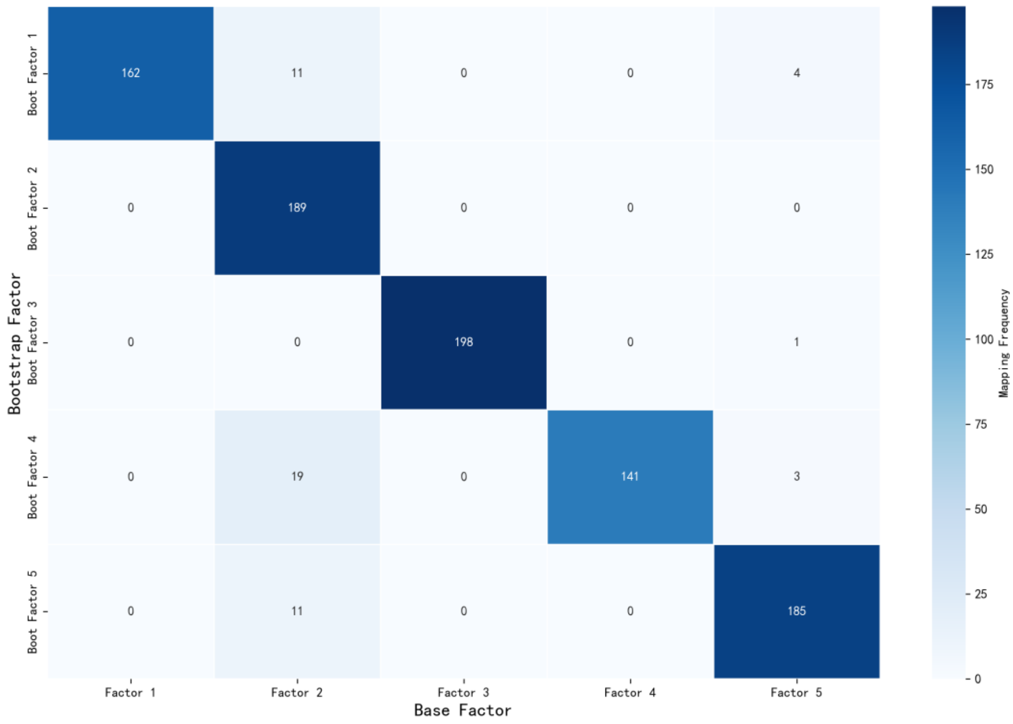

Figure S2. BS Mapping results for bivalves.

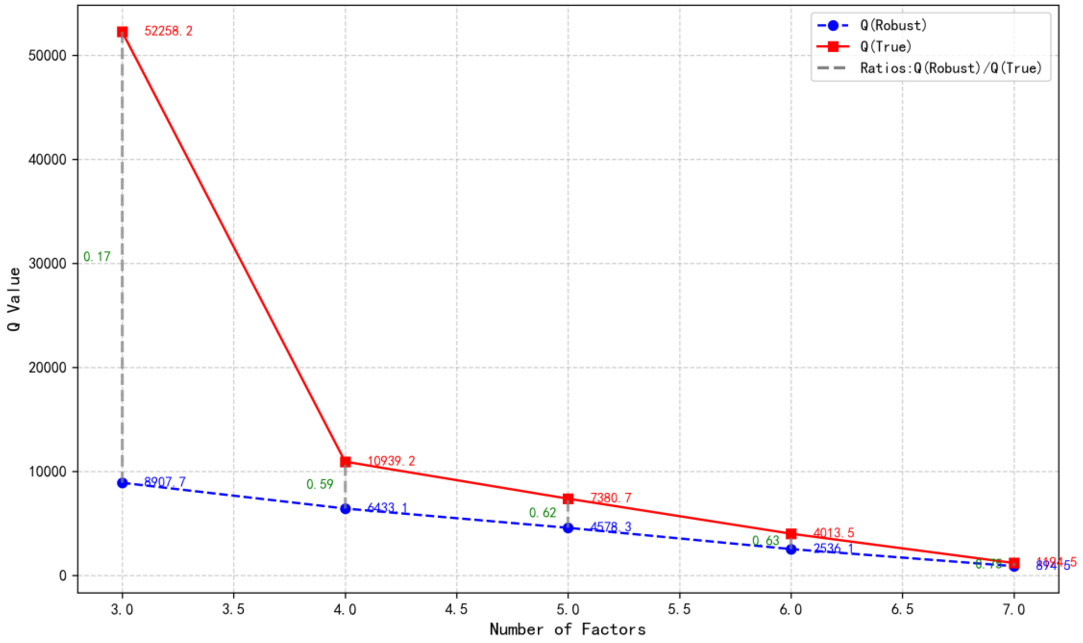

Figure S3. Q-value variation plot for crustaceans, with blue representing Q(Robust), red representing Q(True), and dashed line indicating Q(Robust)/Q(True).

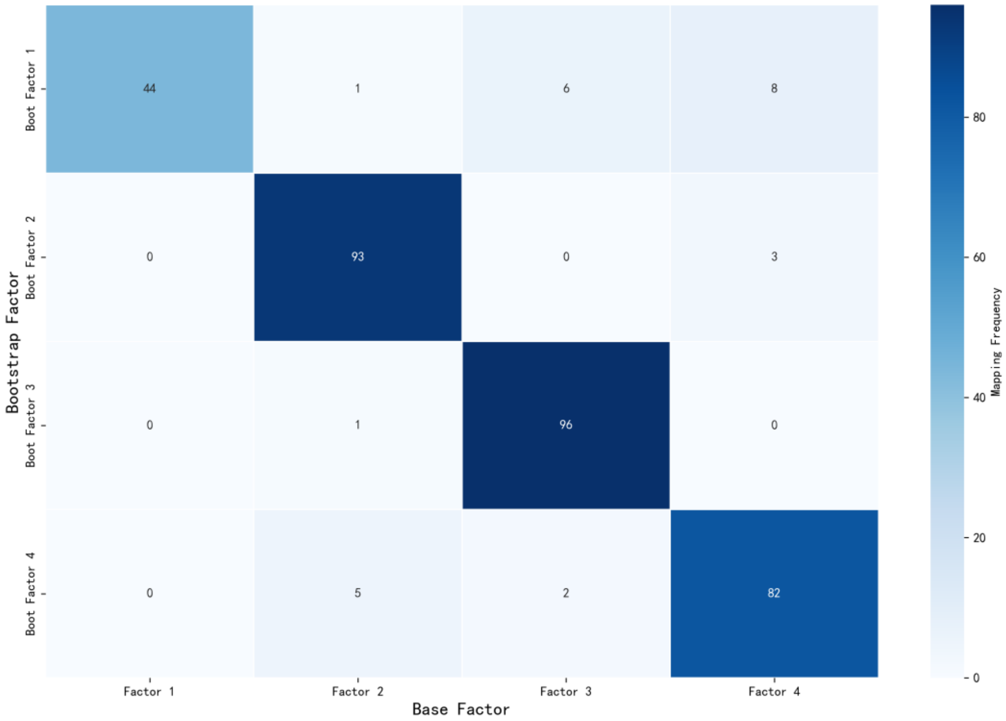

Figure S4. BS Mapping results for crustaceans.

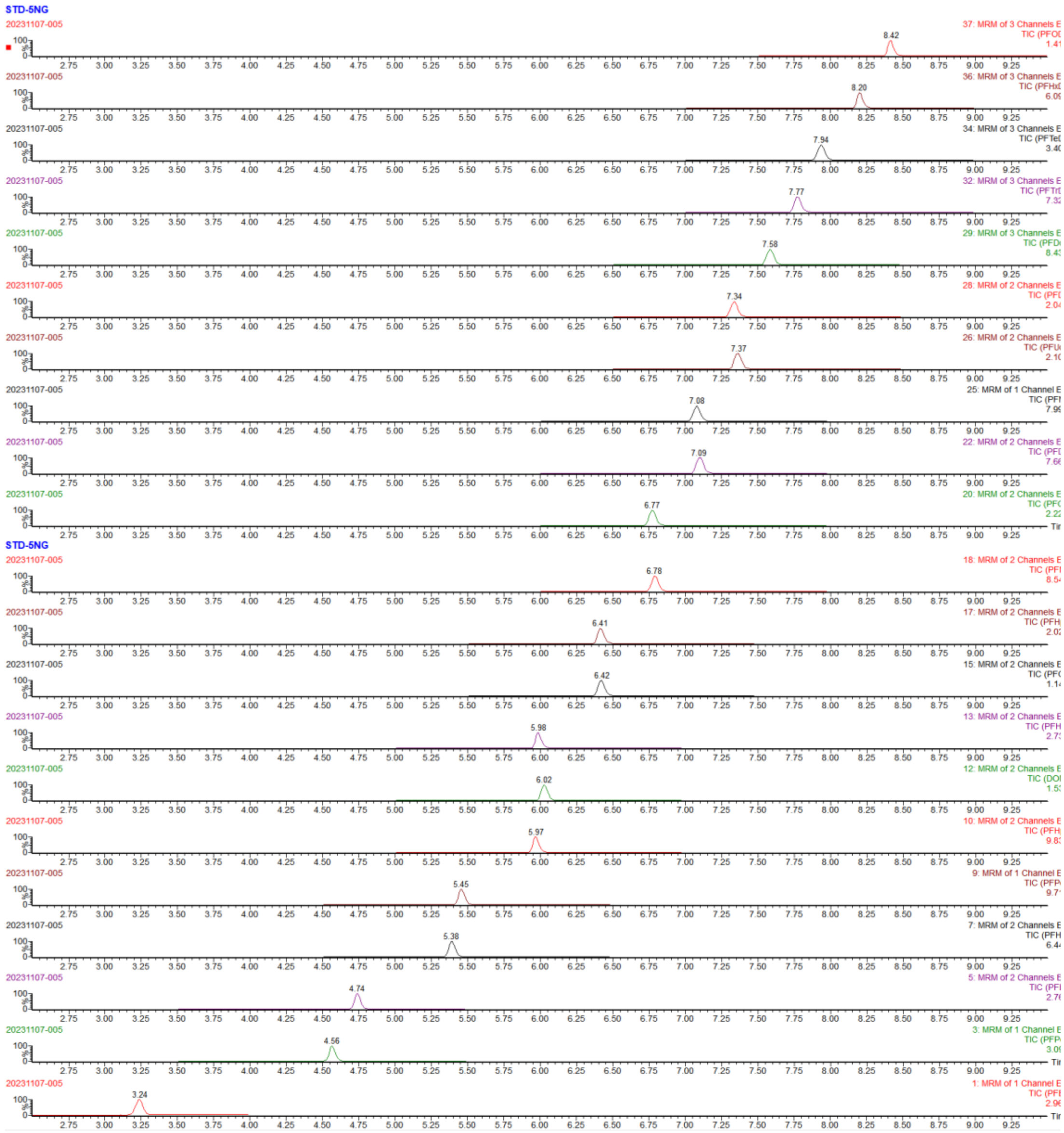

Figure S5. 5ppb standard TIC plot.

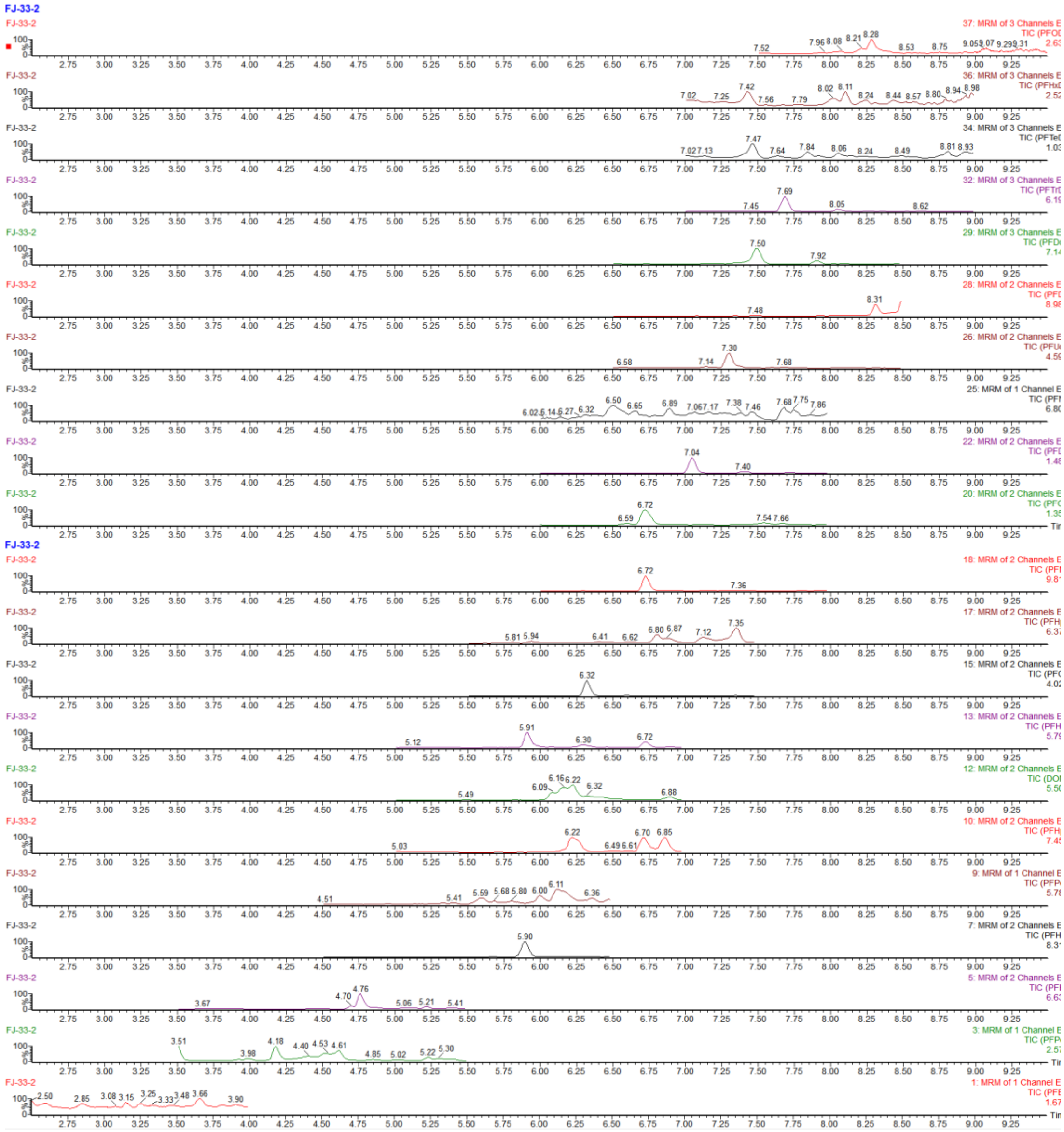

Figure S6. A bivalve shellfish sample's TIC diagram.

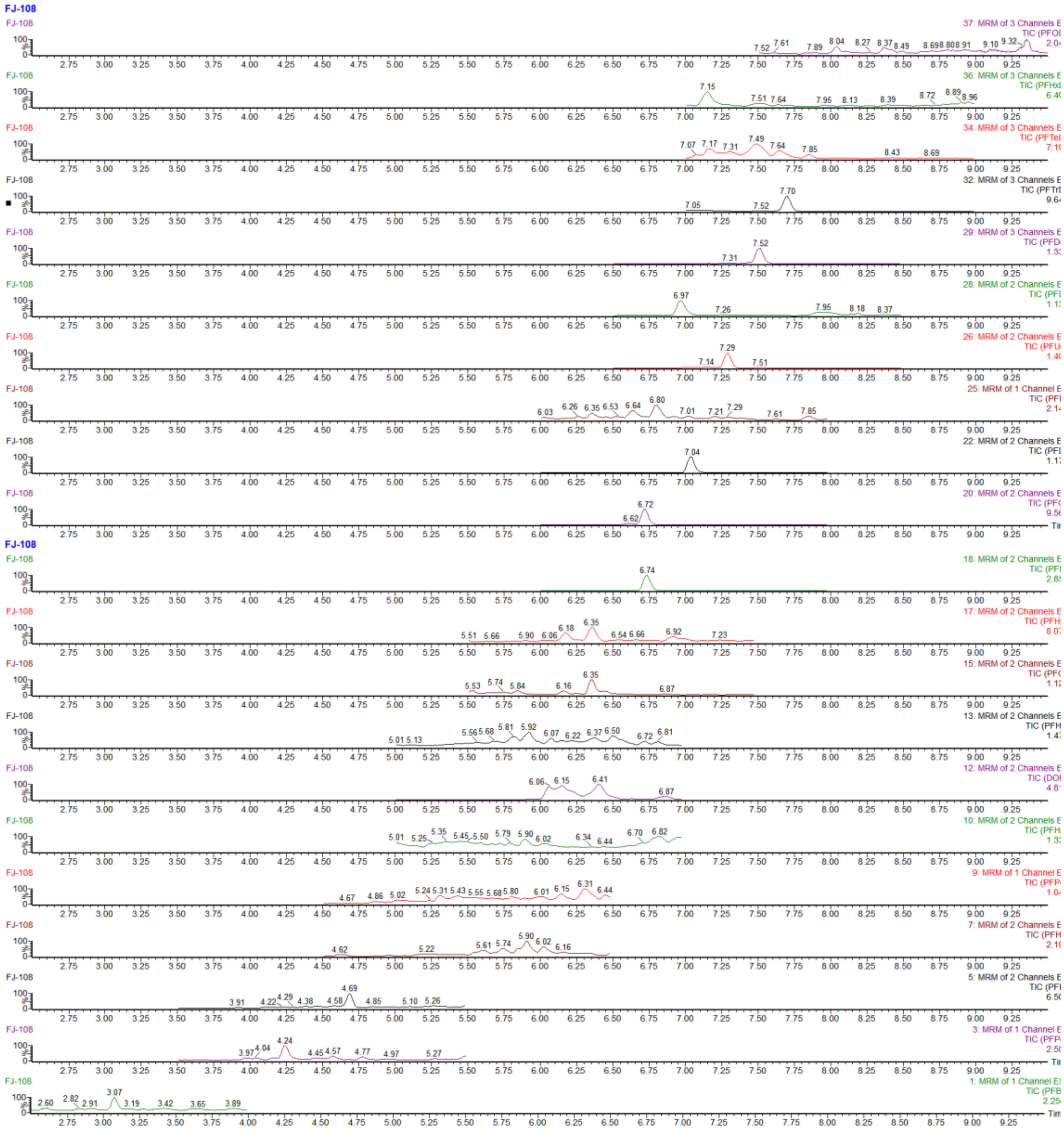

Figure S7.A crustacean sample's TIC diagram.

References

1. Anderson, M.J. Permutational Multivariate Analysis of Variance (PERMANOVA). *Wiley StatsRef: Statistics Reference Online* **2017**, 1–15, doi:10.1002/9781118445112.stat07841.
2. Rivera, J.D.D.; Davies, G.M.; Jahn, W. Flammability and the Heat of Combustion of Natural Fuels: A Review. *Combust. Sci. Technol.* **2012**, *184*, 224–242, doi:10.1080/00102202.2011.630332.
3. Claudet, J.; Pelletier, D.; Jouvenel, J.-Y.; Bachet, F.; Galzin, R. Assessing the Effects of Marine Protected Area (MPA) on a Reef Fish Assemblage in a Northwestern Mediterranean Marine Reserve: Identifying Community-Based Indicators. *Biol. Conserv.* **2006**, *130*, 349–369, doi:10.1016/j.biocon.2005.12.030.
